# Association Between Patatin-Like Phospholipase Domain Containing 3 Gene (PNPLA3) Polymorphisms and Nonalcoholic Fatty Liver Disease: A HuGE Review and Meta-Analysis

**DOI:** 10.1038/srep09284

**Published:** 2015-03-20

**Authors:** Renfan Xu, Anyu Tao, Shasha Zhang, Youbin Deng, Guangzhi Chen

**Affiliations:** 1 Department of Medical Ultrasound, Tongji Hospital, Tongji Medical College, Huazhong University of Science and Technology, Wuhan, People's Republic of China; 2 Department of Internal Medicine and Gene Therapy Center, Tongji Hospital, Tongji Medical College, Huazhong University of Science and Technology, Wuhan, People's Republic of China

## Abstract

We conducted a meta-analysis to assess the association between patatin-like phospholipase domain-containing 3 (PNPLA3) rs738409 polymorphism and nonalcoholic fatty liver disease (NAFLD) and its subtypes simple steatosis(SS) and nonalcoholic steatohepatitis (NASH). The study-specific odds ratios (ORs) and 95% confidence intervals (CIs) were calculated using fixed-effects or random-effects models, with assessment for heterogeneity and publication bias. Twenty-three case-control studies involving 6071 NAFLD patients and 10366 controls were identified. The combined results showed a significant association between NAFLD risk and the rs738409 polymorphism in all genetic models (additive model: OR = 3.41, 95% CI = 2.57–4.52; P < 0.00001). In addition, evidence indicated that the rs738409 polymorphism was significantly associated with NASH in all genetic models (additive model: OR = 4.44, 95% CI = 3.39–5.82; P < 0.00001). The subgroup and sensitivity analyses showed that these changes were not influenced by the ethnicities and ages of subjects or by the source of controls. The rs738409 polymorphism was only significantly associated with risk of simple steatosis in the allele contrast and had no effect in the other genetic models. These findings suggest that the rs738409 polymorphism in PNPLA3 gene confers high cross-ethnicity risk for NAFLD and NASH development.

Nonalcoholic fatty liver disease (NAFLD) is the most common cause of liver disease in western countries, affecting up to 20–35% of the general population^1^, and has emerged as a major public health issue worldwide^2^,^3^. NAFLD has a broad spectrum of manifestations and can be histologically subdivided into simple steatosis and non-alcoholic steatohepatitis (NASH), which include steatosis, lobular inflammation and hepatocyte ballooning with or without fibrosis^4^. Although simple steatosis is generally considered to have a benign hepatological prognosis, NASH much more frequently progresses to fibrosis, cirrhosis and hepatocellular carcinoma in later years^5^,^6^ and will be the leading cause of liver transplantation in the United States by 2020^7^. The precise mechanism responsible for the development and progression of NAFLD has not been elucidated. Some NAFLD patients will progress into NASH with cirrhosis, whereas others do not develop beyond simple steatosis. Currently, there is increasing evidence that genetic^8^,^9^,^10^ as well as environmental factors^11^ play important roles in the progression of NAFLD.

The human patatin-like phospholipase-3 (PNPLA3) gene is localized on human chromosome 22. The PNPLA3 protein, which is also known as adiponutrin, is expressed in both adipocytes and hepatocytes^12^. PNPLA3 exhibits lipase activity against triglycerides and acylglycerol transacetylase activity, and its expression is highly responsive in energy mobilization and the storage of lipid droplets^13^. The PNPLA3 gene is one of the potential candidate genes currently related to NAFLD susceptibility. In 1998, Romeo et al. noted that a single nucleotide polymorphism in residue 148 (I148 M, rs738409), which exhibits a C-to-G transition resulting in an amino acid substitution of isoleucine to methionine, was a strong genetic determinant of NAFLD^10^. Consistent with this result, some following studies also demonstrated an association between the rs738409 polymorphism and NAFLD risk^14^,^15^,^16^. However, it is unclear whether this polymorphism is associated with simple steatosis only or also associated with NASH. Further studies have also attempted to analyze the association between the rs738409 polymorphism and histological parameters of NAFLD^17^,^18^,^19^, but the results are not consistent, partially because only few studies with a limited number of subjects analyzed the association between the rs738409 polymorphism and NASH or simple steatosis.

There is no approved therapy for NAFLD, and the diagnosis of NASH can only be proven by liver biopsy. In addition, it is important to establish whether the associations differ between different subgroups of NAFLD. To clarify the association between the rs738409 polymorphism and risk of NAFLD, we conducted a systematic review and meta-analysis of the available prospective studies with the specific aims of analyzing NAFLD subgroups, including simple steatosis or NASH, to clarify whether the association differed by histological parameters.

## Methods

### Search strategy

We conducted an electronic search of the PubMed, EMBASE and Web of Science databases from their inception until December 2014 to identify the association between the rs738409 polymorphism and NAFLD risk using the following search terms: PNPLA3 and (polymorphism or variant or variation) and (NAFLD or NASH or (non-alcoholic fatty liver disease) or (fatty liver) or steatohepatitis). Additional studies not captured by our database search were identified by surveying the references of the originally identified reviews and research reports and by using the MEDLINE option “Related Articles”. The search was confined to human studies without country restrictions. In addition, the publication language was restricted to English.

### Inclusion and exclusion criteria

Potentially relevant studies were selected based on the following inclusion criteria: (1) studies concerning the association between the *PNPLA3* rs738409 polymorphism and risk of NAFLD; (2) case-control studies based on unrelated individuals; (3) studies in which the diagnosis of NAFLD was clear; (4) studies that provide the number of NAFLD cases and controls and the frequency of the rs738409 genotypes; and (5) studies published in English. The major reasons for study exclusion were the following: (1) case-only study or overlapping data; (2) studies with a sample size less than one hundred; (3) studies with abstracts only and reports published as comment and review papers; and (4) studies with secondary causes of steatosis, including alcohol abuse, the use of drugs, surgical procedures and hepatitis B and hepatitis C virus infection.

### Data extraction

Two investigators independently selected the trials and extracted the data, and disagreements or uncertainties were resolved by consensus. The following data were extracted: first author, publication year, country of origin, ethnicity of studied population, sex ratio, mean age, diagnostic criteria for NAFLD, number of individuals in the case and control groups, frequency of PNPLA3 genotypes in the cases and controls; and consistency with the Hardy-Weinberg equilibrium(HWEs).

### Study quality assessment

The quality of the studies was assessed independently by two investigators according to the quality assessment scores developed from the genetic association studies conducted by Thakkinstian et al. The total scores ranged from 0 (worst) to 13 (best)^20^. The criteria of the quality assessment used to analyze the studies in this meta-analysis are available in Table S1.

### Statistical analysis

The strength of the association between the PNPLA3 polymorphism and NAFLD risk was assessed by the odds ratios (ORs) and 95% confidence interval (CI). The Chi-square test was used to assess the Hardy-Weinberg equilibrium (HWE) in order to analyze the genotype distribution in the control groups. Meta-analyses were performed for four genotype contrasts per outcome: allele contrast (G versus C), dominant model (GG+CG versus CC), recessive model (GG versus CG+CC), and additive model (GG versus CC)^21^,^22^. The Cochrane Q statistic and the inconsistency index (I^2^) were used to calculate the heterogeneity among the studies, and a P value < 0.10 or I^2^ > 50% was considered to be significant^23^. If heterogeneity existed among the studies, the random-effect model (the Dersimonian and Laird method) was used to calculate the pooled OR. Otherwise, a fixed-effect model (the Mantel-Haenszel method) was used for outcomes without obvious heterogeneity^24^. Sensitivity analyses were performed to assess the stability of the results by excluding one study at a time in order to analyze the influence of each study on the overall OR. The publication bias was assessed using funnel plots and Egger's test^25^.

Three subgroup analyses were additionally carried out by ethnicity (Caucasian, Asian or Hispanics), mean age (pediatric or adult) and source of the controls (hospital based or population based). The statistical analysis was performed with RevMan software version 5 (Cochrane Collaboration) and STATA software version 10.0 (Stata Corporation). A P value < 0.05 was considered to be statistically significant in this trial unless otherwise specified.

## Results

### Literature search

**Figure 1:**
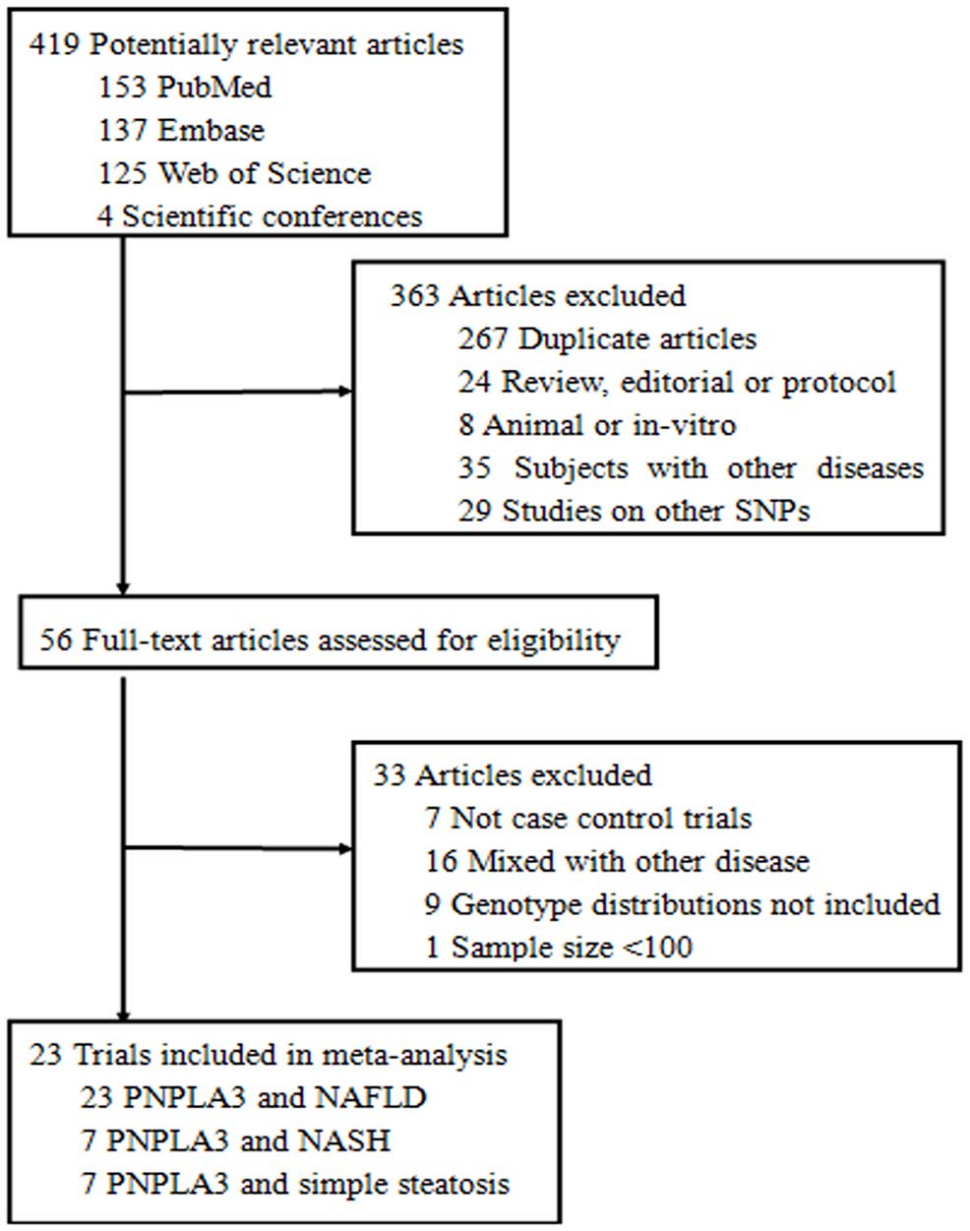
Flowchart of the study selection.

The search strategy initially identified 419 potentially relevant articles, and 363 articles were determined to be irrelevant after a review of the titles and abstracts. Thus, 56 trials proceeded to a full-text review, and an additional 33 studies were excluded. Finally, 23 articles were ultimately selected for inclusion in the meta-analysis^17^,^18^,^19^,^26^,^27^,^28^,^29^,^30^,^31^,^32^,^33^,^34^,^35^,^36^,^37^,^38^,^39^,^40^,^41^,^42^,^43^,^44^,^45^. A flow describing the article selection process for this meta-analysis is shown in Figure 1. Of all of the studies included, 10 studies involved Caucasians^17^,^19^,^26^,^27^,^28^,^29^,^30^,^31^,^32^,^42^, 12 studies investigated Asians^18^,^33^,^34^,^35^,^36^,^37^,^38^,^39^,^40^,^43^,^44^,^45^, and 1 study researched Hispanics^41^. All of the studies followed a case-control design, 8 studies used population-based controls^16^,^18^,^28^,^33^,^40^,^41^,^42^,^43^, and 15 studies used hospital-based controls^17^,^19^,^26^,^29^,^30^,^31^,^32^,^34^,^35^,^36^,^37^,^38^,^39^,^44^,^45^. In addition, 19 studies were conducted in adult patients^16^,^17^,^18^,^19^,^26^,^28^,^29^,^30^,^31^,^32^,^34^,^35^,^36^,^37^,^38^,^39^,^42^,^44^,^45^, and 4 investigated pediatric patients^33^,^40^,^41^,^43^. The distribution of genotypes in the controls was consistent with HWE in 21 studies^17^,^18^,^19^,^26^,^27^,^29^,^31^,^32^,^33^,^34^,^35^,^36^,^37^,^38^,^39^,^40^,^41^,^42^,^43^,^44^,^45^ and insufficient in the 2 other studies^28^,^30^. The quality score of the included studies ranged from 7 to 11 (Table S1). The characteristics of the included studies are presented in Table 1.

### Association between rs738409 and risk for NAFLD

#### All studies

**Figure 2:**
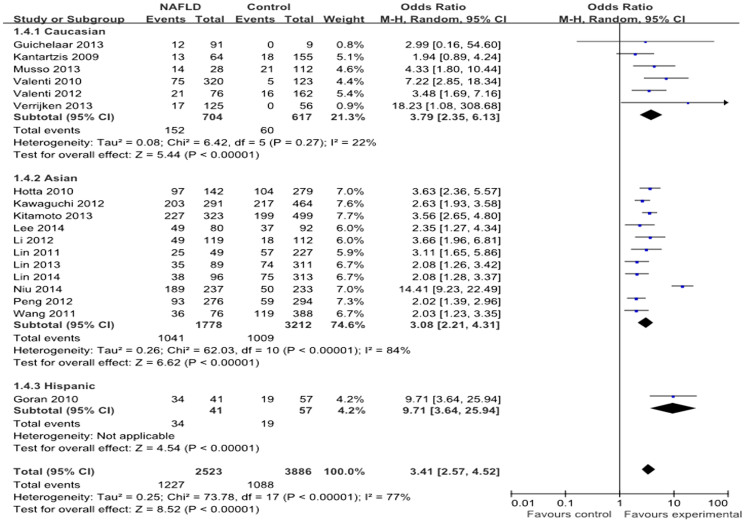
Forest plot of NAFLD susceptibility associated with rs738409 polymorphism at additive model (GG vs CC).

A total of 23 studies with 6071 cases and 10366 controls reported an association between the rs738409 polymorphism and NAFLD risk^17^,^18^,^19^,^26^,^27^,^28^,^29^,^30^,^31^,^32^,^33^,^34^,^35^,^36^,^37^,^38^,^39^,^40^,^41^. Overall, the frequency of the G allele was 49.5% in NAFLD and 34.8% in the controls. The Hispanic population bears the highest frequency of the G allele (69.0% cases vs. 41.9% controls), followed by the Asian (54.2% cases vs. 39.9% controls) and Caucasian (42.2% cases vs. 22.7% controls) populations. The distribution of the rs738409 genotypes and alleles is presented in Table 2. Strong evidence of an association between the rs738409 polymorphism and NAFLD risk was found in all genetic models: allele contrast (OR = 2.10, 95% CI = 1.78–2.48, P < 0.00001; heterogeneity test: I^2^ = 89%, P < 0.00001); dominant model (OR = 2.06, 95% CI = 1.75–2.43, P < 0.00001; heterogeneity test: I^2^ = 67%, P < 0.00001); recessive model (OR = 2.49, 95% CI = 2.01–3.08, P < 0.00001; heterogeneity test: I^2^ = 72%, P < 0.0001); and additive model (OR = 3.41, 95% CI = 2.57–4.52, P < 0.00001; heterogeneity test: I^2^ = 77%, P < 0.00001) (Figure 2). After exclusion of the two articles deviating from HWE in the cases and controls, the results of the relationship was not influenced significantly in all genetic models (Table 3).

#### Subgroup analyses

Subgroup analyses were conducted to explore the differences between ethnicity, mean age and sources of the controls. In the subgroup analysis by ethnicity, significant association was found between the rs738409 polymorphism and NAFLD risk among the Caucasian, Asian and Hispanic populations. The association between rs738409 polymorphism and NAFLD was most significant in Hispanic population, which followed by Caucasian population, and the association was weakest in Asian population. The analyses also showed that the risk of NAFLD was significantly increased in both adult participants and pediatric subjects. In addition, the G allele was strongly associated with NAFLD susceptibility in hospital-based controls and population-based controls. The results were consistent in all genetic models. More details are presented in Table 3.

### Histological Severity of NAFLD

**Figure 3:**
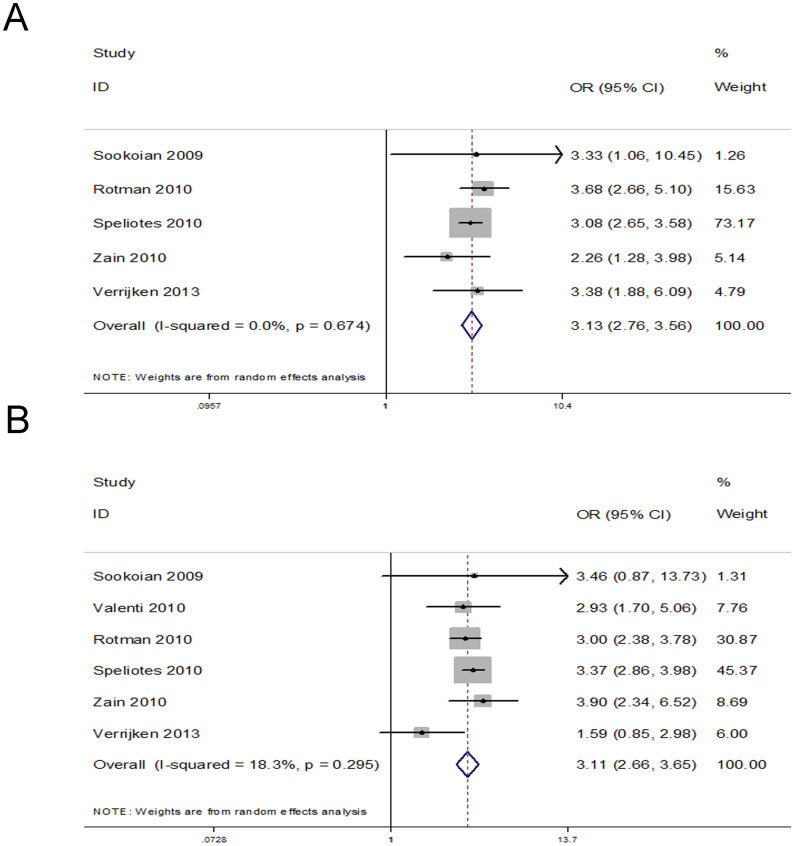
(A)The forest plot for the association between rs738409 polymorphism and the risk of necroinflammation (additive model: GG vs CC). (B)The forest plot for the association between rs738409 polymorphism and the risk of fibrosis (additive model: GG vs CC).

Five eligible studies were used to investigate the association between the rs738409 polymorphism and lobular necroinflammation, including 1978 patients. A statistically significant association was seen between carrying GG genotype and higher inflammation scores (OR = 3.13, 95% CI = 2.76–3.56, P < 0.00001; heterogeneity test: I^2^ = 0%, P = 0.674) with obvious publication bias (Egger test: P = 0.980) (Figure 3). The six eligible studies with 2552 patients analyze the relationship between rs738409 polymorphism and fibrosis. The analysis pointed out that the GG genotype was significantly associated with fibrosis score (OR = 3.11, 95% CI = 2.66–3.65, P < 0.00001; heterogeneity test: I^2^ = 18.3%, P = 0.295) and the publication bias was not significant (Egger test: P = 0.457) (Figure 3).

### Association between rs738409 and risk for simple steatosis

**Figure 4:**
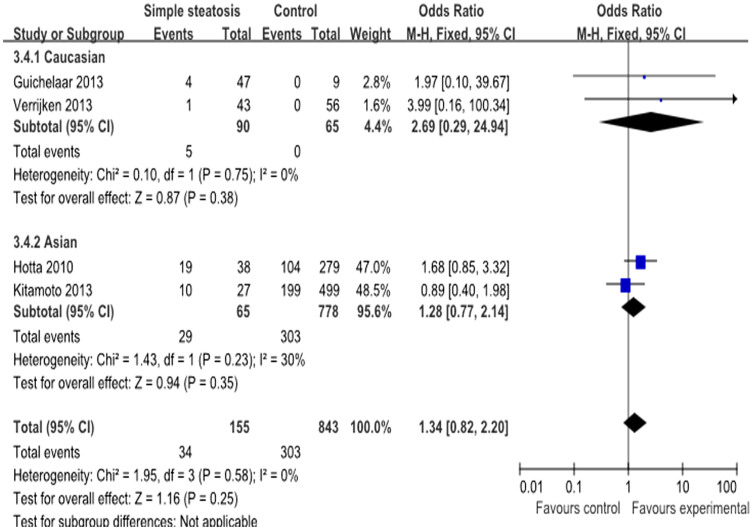
Forest plot of simple steatosis susceptibility associated with rs738409 polymorphism at additive model (GG vs CC).

Overall, 7 studies with 387 cases and 2306 controls analyzed the rs738409 polymorphism and risk of simple steatosis^17^,^18^,^19^,^28^,^32^,^34^,^36^. Interestingly, the frequency of the risk G allele was very close between the cases (38.1%) and controls (38.0%). In Caucasian subjects, the frequency of the G allele was 34.3% in cases and 23.2% in controls, and these values are lower than those found in the Asian population (44.3% cases vs. 42.3% controls) (Table 4). We analyzed the relationship between the G allele and the risk of simple steatosis. No significant association was observed between rs738409 polymorphism and simple steatosis under additive model (OR = 1.34, 95% CI = 0.82–2.20, P = 0.25; heterogeneity test: I^2^ = 0%, P = 0.58), dominant model and recessive model (Figure 4). However, a significant association was found in allele contrast. A further subgroup analysis based on ethnicity showed no obvious association between the rs738409 polymorphism and simple steatosis in Asian subjects, while a strong association was found in the Caucasian population under the allele contrast instead of the other three genetic models. (Table 5).

### Association between rs738409 and NASH risk

**Figure 5:**
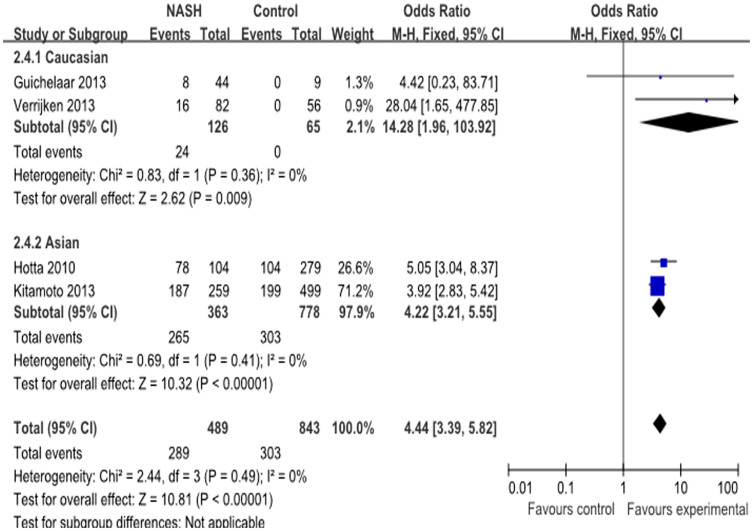
Forest plot of NASH susceptibility associated with rs738409 polymorphism at additive model (GG vs CC).

Overall, seven studies with 1466 cases and 2306 controls reported the rs738409 polymorphism and risk of NASH^17^,^18^,^19^,^28^,^32^,^34^,^36^: four studies conducted in Caucasians and three studies performed in Asian populations. The pooled overall frequency of the risk G allele was 53.5% in the cases and 38.0% in the controls. The G allele varied widely between the different populations: high in the Asian populations (60.9% cases vs.42.3% controls) and lower in the Caucasian subjects (45.9% cases vs. 23.2% controls) (Table 4). Strong evidence of an association was detected between the rs738409 polymorphism and NASH risk under the additive model (OR = 4.44, 95% CI = 3.39–5.82, P < 0.00001; heterogeneity test: I^2^ = 0%, P = 0.49) (Figure 5). The association was also significant in the other three genetic models, and no evidence of heterogeneity was observed between the studies. Evidence of a strong association between the rs738409 polymorphism and NASH susceptibility was also found in both Asian and Caucasian populations with all genetic models. In addition, Caucasian populations with rs738409 polymorphism are more easily develop into NASH than Asian populations. The results are described in Table 6.

### Sensitivity and Publication Bias

**Figure 6:**
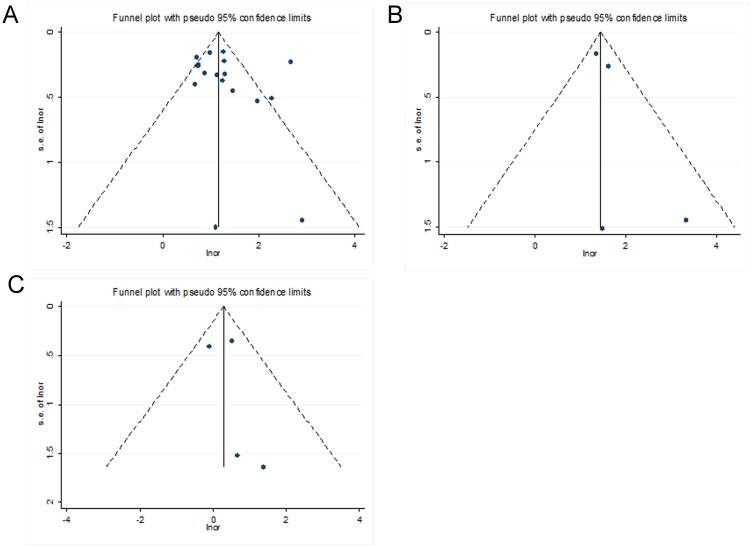
Publication bias on rs738409 polymorphism under additive model. (A) Funnel plot of studies of the rs738409 variant and NAFLD. (B) Funnel plot of studies of the rs738409 variant and simple steatosis. (C) Funnel plot of studies of the rs738409 variant and NASH.

Sensitivity analysis was performed under additive model to evaluate the influence of a specific study on the overall estimate. The corresponding pooled ORs with 95% CIs produced similarly before and after omitting each study at a time, indicating that our results were stable and reliable (Table S2). The funnel plots of the studies were symmetric in the current meta-analysis (Figure 6). Furthermore, the results of Egger's test did not support the existence of publication bias (additive model: NAFLD: P = 0.467; SS: P = 0.611; NASH: P = 0.282).

## Discussion

The current meta-analysis provided a systematic assessment of the association between the PNPLA3 rs738409 polymorphism and susceptibility to NAFLD, including its subtypes simple steatosis and NASH. Our results suggested that rs738409 polymorphism exerted a significant influence not only on NAFLD risk, but also on histological severity of NAFLD. In addition, a further analysis showed that individuals with the rs738409 polymorphism experienced a significantly increased risk for NASH. However, our meta-analysis did not show a definite association of rs738409 polymorphism with simple steatosis.

Our results are consistent with those from a previous meta-analysis conducted by Sookoian et al.^14^, which showed a significant association between the rs738409 polymorphism and NAFLD (OR = 3.26, 95% CI = 2.73–3.89, P < 0.00001) and a significant association between the rs738409 polymorphism and NASH (OR = 3.26, 95% CI = 2.14–4.95, P < 0.00001), similar to the results reported in this manuscript. In the present meta-analysis, analysis of the rs738409 polymorphism revealed a significantly increased NAFLD risk in all genetic models. When the data were stratified by subject ethnicity, a significant correlation was found in all three populations, suggesting that the susceptibility genes may be a strong indicator across different races. In the population-based and hospital-based control studies, a significant correlation was also observed in all genetic models, suggesting that our results were not influenced by the source of controls. In addition, the association between the rs738409 polymorphism and NAFLD risk was also significant in both adult and children populations, indicating that the results are highly stable and not influenced by ethnicity, source of the controls and age of participants.

A large population-based study that involved 9229 multiethnic population, including African-Americans, Hispanics and European-Americans, revealed that patients with the rs738409 polymorphism are associated with a higher risk of NAFLD compared with normal controls^10^. These findings are generally consistent with individual published reports because 70–90% of the trials showed an association between the rs738409 polymorphism and NAFLD risk^27^,^38^,^41^. The underlying mechanism for how PNPLA3 genotype increases NAFLD susceptibility remains to be elucidated. The questions that have been raised are whether the I148M polymorphism increases liver damage favoring the accumulation of fatty acids in lipid droplets or increases the susceptibility to progress into NASH and fibrogenesis.

It should be noted that rs738409 polymorphism was only significantly associated with increased simple steatosis risk under allelic model, but not under the other three genetic models. When stratified by ethnicity, we only detect a significant association in the Asian subgroup under allele contrast, but failed to detect a significant association in the Caucasion population under all genetic models. This meta-analysis of the associations of the rs738409 polymorphism with NASH showed a significant relation. In the subgroup analysis stratified by ethnicity, similar correlations were observed in both Caucasian and Asian populations. The results from the allele contrast were consistent with those from the other genetic models. The sensitivity analysis revealed that no single study qualitatively changed the pooled odds ratios. These findings suggested that rs738409 polymorphism was strongly associated with NASH.

In our meta-analysis, it appears that the rs738409 polymorphism is more likely to increase the NASH risk instead of simple steatosis. Consistent with our results, animal studies have revealed that, although PNPLA3 has triglyceride lipase activity and is responsible for the transalkylation of acylglycerol, knockout of PNPLA3 has no effect on liver steatosis or insulin resistance^46^. Further epidemiological studies have also noted that this G allele variation did not affect the main risk factors for steatosis, including insulin resistance, LDL, HDL, total cholesterol and glucose levels^29^. Other polymorphisms, such as CD14 rs2569190 and GCLC rs4140528, are also regarded to increase the risk of NASH instead of simple steatosis^47^. There are some possible reasons to explain this phenomenon. First, the effect of the rs738409 G allele may be involved in the differential expression and function of variant PNPLA3 instead of resulting in a loss of function of the wild-type protein. Second, there may be some gene-gene interactions. It is possible that the difference in phenotypes may be caused by some other genetic variant that is strongly linked to rs738409. Third, although NASH and simple steatosis are currently regarded as two histological subtypes along the unique spectrum of NAFLD, evidence suggests that these two conditions may be not only different from the histological syndrome but also varied from pathophysiological standpoints. The results that the association between NASH risk and the rs738409 genotype is independent of simple steatosis might suggest that simple steatosis may not be the essential condition for the progressive damage. Simple steatosis and NASH are likely to be two independent conditions in the NAFLD spectrum.

Despite the inevitable limitations of this meta-analysis, we believe that our research provides useful information. First, the individual sample size of each study included in our meta-analysis was too small to obtain a definite association between rs738409 polymorphisms and NAFLD risk, but the pooled odds ratios generated from the 23 studies significantly increased the statistical power of the analysis compared to that obtained with a single study. Moreover, the protocol of this meta-analysis has been well-designed with explicit criteria and methods for study selection, data extraction and data analysis, which allowed reliable inferences about causality. Third, there was no significant publication bias in this meta-analysis, and the results of the sensitivity analysis support the stability of the results.

However, some limitations of this meta-analysis should be addressed. First, the retrieved literature may not be sufficiently comprehensive. Only published case-control studies were included in this meta-analysis. Second, most of the study subjects were of Caucasian and Asian ancestry, and the Hispanic subgroup was very limited in this meta-analysis. Thus, potential selective bias and publication bias may have occurred. Third, because NAFLD was a multifactor disease, the potential effects of gene-gene and gene-environment interactions should be considered. Fourth, the sample size of NASH in this meta-analysis was so small that the statistical power for making a definitive conclusion regarding the possible risk of the rs738409 polymorphism was limited.

In conclusion, results from this meta-analysis showed that the G allele at PNPLA3 gene was a risk factor for NAFLD and its subtype NASH, especially in Asian, Caucasian and Hispanic populations. However, no association was observed between the rs738409 polymorphism and simple steatosis risk. Further studies with higher quality, more participants and various ethnicities are needed to obtain a more precise estimate of the genetic effects.

## Author Contributions

R.-F.X. and G.-Z.C. conceived the study design, and wrote the manuscript; A.-Y.T., S.-S.Z. and Y.-B.D. performed the analyses. All authors read and approved the final manuscript.

## Supplementary Material

Supplementary Information Supplementary information

## Figures and Tables

**Table 1:** Characteristics of Studies Included in this meta-analysis

Author	Year	Country or Region	Ethnicity	Source of Control	Genotyping method	Age	Female n (%)	NAFLD diagnosis	Liver Biopsy(n)	Cases	Controls	Quality Score
Kantartzis et al.	2009	Germany	Caucasian	H-B	TaqMan	Adult	200(60.6)	H-MRS	NA	105	225	10
Sookoian et al.	2009	Argentina	Caucasian	H-B	Allele specific PCR	Adult	186(69.9)	US and LB	103	172	94	10
Valenti et al.	2010	Italy/United Kingdom	Caucasian	P-B	TaqMan	Adult	Italy: 114(26.3) UK: 123 (38)	LB	574	574	179	11
Rotman et al.	2010	USA	Caucasian	P-B	MassARRAY Sequenome	Adult	NA	LB	766	120	766	7
Speliotes et al.	2010	USA	Caucasian	H-B	MassARRAY Sequenome	Adult	NA	LB	678	678	1405	10
Goran et al.	2010	USA	Hispanic	P-B	TaqMan	Pediatric	129 (68.6)	DEXA	NA	71	188	9
Lin et al.	2010	Taiwan	Asian	P-B	TaqMan	Pediatric	174 (33.5)	US	NA	102	418	11
Hotta et al.	2010	Japan	Asian	H-B	TaqMan	Adult	527 (63.4)	LB	253	253	575	8
Wang et al.	2011	Taiwan	Asian	H-B	TaqMan	Adult	472 (53.7)	US	NA	156	723	10
Petit et al.	2011	France	Caucasian	H-B	Real-time PCR	Adult	120 (51.3)	H-MRS	NA	149	85	8
Zain et al.	2012	Malaysia	Asian	H-B	TaqMan	Adult	180 (52.6)	LB	144	144	198	10
Kawaguchi et al.	2012	Japan	Asian	H-B	BeadChip	Adult	741 (50.7)	LB	529	529	932	10
Valenti et al.	2012	Italian	Caucasian	H-B	Real-time PCR	Adult	87 (21.7)	LB	144	144	257	9
Li et al.	2012	China	Asian	H-B	TaqMan	Adult	NA	US	NA	203	202	10
Peng et al.	2012	China	Asian	H-B	MassARRAY Sequenome	Adult	308 (27.8)	US	NA	553	553	11
Lin et al.	2013	Taiwan	Asian	P-B	TaqMan	Pediatric	237 (30.3)	US	NA	182	599	9
Guichelaar et al.	2013	USA	Caucasian	H-B	TaqMan	Adult	122 (84.7)	LB	144	132	12	8
Verrijken et al.	2013	Belgium	Caucasian	H-B	TaqMan	Adult	331 (70.4)	LB	287	208	79	10
Kitamoto et al.	2013	Japan	Asian	P-B	BeadChip	Adult	782 (49.6)	LB	564	564	1946	11
Musso et al.	2013	Italy	Caucasian	P-B	TaqMan	Adult	78 (36.8)	US	NA	51	161	11
Lin et al.	2014	Taiwan	Asian	P-B	TaqMan	Pediatric	242 (30.4)	US	NA	191	606	11
Niu et al.	2014	China	Asian	H-B	ABI Sequencer	Adult	426 (53.3)	US	NA	390	409	10
Lee et al.	2014	Korea	Asian	H-B	TaqMan	Adult	178 (52.5)	US	NA	155	184	11

P-B, population-based study; H-B, hospital-based study; H-MRS: hydrogen magnetic resonance (H-MR) spectroscopy, US: liver ultrasonographic examination, LB: liver biopsy, DEXA: dual energy-ray absorptiometry, NA: not available.

**Table 2:** The distribution of alleles and genotypes of PNPLA3 in NAFLD studies

	Sample size		Genotype in cases			Genotype in controls			Case		Control		G allele (%)		C allele (%)		
First Author	Cases	Controls	GG	CG	CC	GG	CG	CC	G	C	G	C	Cases	Controls	Cases	Controls	HWE P value
Kantartzis	105	225	13	41	51	18	70	137	67	143	106	344	31.9	23.6	68.1	76.4	YES
Sookoian	103	94	NA	NA	NA	NA	NA	NA	130	76	63	125	63.1	33.6	36.9	66.4	YES
Valenti 2010	574	179	75	254	245	5	56	118	404	744	66	292	35.2	18.4	64.8	81.6	0.59
Rotman	520	336	NA	NA	NA	NA	NA	NA	516	524	153	519	49.6	22.8	50.4	77.2	NA
Speliotes	592	1405	NA	NA	NA	NA	NA	NA	592	592	618	2192	50	22.0	50	78.0	YES
Goran	71	188	34	30	7	19	60	38	98	44	98	136	69	26	31	74	0.56
Lin 2011	102	418	26	52	24	59	192	167	104	100	310	526	51.0	37.1	49.0	62.9	0.75
Hotta	253	575	175	97	111	104	296	175	305	201	504	646	88.3	43.8	11.7	56.2	0.28
Wang	156	723	40	80	36	269	335	119	152	160	573	873	51.3	60.4	48.7	39.6	0.40
Petit	149	85	NA	NA	68	NA	NA	51	NA	NA	NA	NA	NA	NA	NA	NA	NA
Zain	144	198	NA	NA	NA	NA	NA	NA	130	158	95	301	45.1	24.0	54.9	76.0	YES
Kawaguchi	529	932	217	468	247	203	236	88	642	412	902	962	85.2	34.4	14.8	65.6	0.17
Valenti 2012	144	257	21	68	55	16	95	146	110	178	127	387	38.2	24.7	61.8	75.3	0.92
Li	203	202	49	84	70	18	90	94	182	224	126	278	44.8	31.0	55.2	69.0	0.59
Peng	553	553	93	276	183	59	259	235	462	642	377	729	41.8	34.1	58.2	65.9	0.32
Lin 2013	182	599	35	93	54	74	288	237	163	201	436	762	44.8	36.4	55.2	63.6	0.35
Guichelaar	132	12	12	41	79	0	3	9	65	199	3	21	24.6	12.5	75.4	87.5	0.62
Verrijken	208	79	17	83	108	0	23	56	117	299	140	434	20.4	5.5	79.6	94.5	0.13
Kitamoto	564	1946	227	241	96	199	513	300	695	433	911	1113	61.6	23.4	38.4	76.6	0.44
Musso	51	161	14	23	14	21	49	91	51	51	91	231	50	28.3	50	71.7	YES
Lin	191	606	38	95	58	75	293	238	171	211	443	769	44.8	36.6	55.2	63.4	0.30
Niu	390	409	189	153	48	50	176	183	531	249	276	542	68.1	33.7	31.9	66.3	0.45
Lee	155	184	49	75	31	37	92	55	173	137	166	202	55.8	45.1	44.2	54.9	0.90

NA: not applicable YES: studies have already pointed out that the data was HWE, but the data was not applicable.

**Table 3:** Association between PNPLA3 polymorphism and NAFLD risk

Subgroup	Inherited model	Study number	NO. of cases/controls(n/n)	P_heterogeneity_	I^2^ (%)	Pooled OR (95%CI)	P value^a^
Total studies	Allele contrast	22	11838/18552	P < 0.00001	89	2.10 (1.78, 2.48)	P < 0.00001
	Dominant model	19	4709/7328	P < 0.0001	67	2.06 (1.75, 2.43)	P < 0.00001
	Recessive model	18	4560/7243	P < 0.0001	72	2.49 (2.01, 3.08)	P < 0.00001
	Additive model	18	2523/3886	P < 0.00001	77	3.41 (2.57,4.52)	P < 0.00001
Studies excluded for DHWE							
	Allele contrast	21	10798/17880	P < 0.00001	89	2.05 (1.74, 2.42)	P < 0.00001
	Dominant model	18	4560/7243	P < 0.00001	69	2.02 (1.84, 2.20)	P < 0.00001
	Recessive model	18	4560/7243	P < 0.00001	72	2.51 (2.28, 2.77)	P < 0.00001
	Additive model	18	2523/3886	P < 0.00001	77	3.32 (2.94, 3.74)	P < 0.00001
Ethnicity							
Caucasian	Allele contrast	9	4858/5496	P < 0.0001	75	2.56 (2.06, 3.18)	P < 0.00001
	Dominant model	7	1363/998	P = 0.60	0	2.21 (1.83, 2.67)	P < 0.00001
	Recessive model	6	1214/913	P = 0.35	10	2.68 (1.78, 4.05)	P < 0.00001
	Additive model	6	704/617	P = 0.27	22	3.79 (2.35, 6.13)	P < 0.00001
Asian	Allele contrast	12	6838/12822	P < 0.00001	88	1.82 (1.52,2.18)	P < 0.00001
	Dominant model	11	3275/6213	P < 0.00001	78	1.95 (1.56, 2.43)	P < 0.00001
	Recessive model	11	3275/6213	P < 0.00001	81	2.33 (1.81, 2.99)	P < 0.00001
	Additive model	11	1778/3212	P < 0.00001	84	3.08 (2.21, 4.31)	P < 0.00001
Hispanics	Allele contrast	1	142/234	NA	NA	3.09 (1.99, 4.80)	P < 0.00001
	Dominant model	1	71/117	NA	NA	4.40 (1.84, 10.51)	P = 0.0009
	Recessive model	1	71/117	NA	NA	4.74 (2.41, 9.33)	P < 0.00001
	Additive model	1	41/57	NA	NA	9.71 (3.64, 25.94)	P < 0.00001
Control source							
Population based	Allele contrast	7	4076/5836	P < 0.00001	90	2.17 (1.60, 2.95)	P < 0.00001
	Dominant model	7	2038/2918	P < 0.00001	82	2.47 (2.14, 2.85)	P < 0.00001
	Recessive model	7	2038/2918	P < 0.00001	83	3.04 (2.00,4.62)	P < 0.00001
	Additive model	7	1139/1542	P < 0.00001	87	4.61 (2.58, 8.23)	P < 0.00001
Hospital based	Allele contrast	15	7762/12716	P < 0.00001	89	2.07 (1.68, 2.54)	P < 0.00001
	Dominant model	12	2671/4410	P = 0.65	0	1.76 (1.57,1.97)	P < 0.00001
	Recessive model	11	2522/4325	P = 0.26	19	2.10 (1.78, 2.47)	P < 0.00001
	Additive model	11	1384/2344	P = 0.34	11	2.62 (2.20, 3.13)	P < 0.00001
Age of participants							
Adult	Allele contrast	18	10746/15072	P < 0.00001	90	2.19 (1.82, 2.62)	P < 0.00001
	Dominant model	15	4163/5588	P < 0.0001	70	2.10 (1.74, 2.54)	P < 0.00001
	Recessive model	14	4014/5503	P < 0.00001	75	2.59 (2.01, 3.34)	P < 0.00001
	Additive model	14	2248/2978	P < 0.00001	79	3.54 (2.54, 4.94)	P < 0.00001
Pediatric	Allele contrast	4	1092/3480	P = 0.01	73	1.73 (1.31, 2.29)	P = 0.0001
	Dominant model	4	546/1740	P = 0.09	54	1.89 (1.34, 2.66)	P < 0.00001
	Recessive model	4	546/1740	P = 0.07	58	2.18 (1.47, 3.22)	P < 0.0001
	Additive model	4	275/908	P = 0.03	66	2.97 (1.75, 5.02)	P < 0.0001

a: Test for overall effect. NA: Not applicable.

**Table 4:** The distribution of alleles and genotypes of PNPLA3 in SS studies and NASH studies

	Sample size		Genotype in cases			Genotype in controls			Case		Control		G allele (%)		C allele (%)	
First Author	Cases	Controls	GG	CG	CC	GG	CG	CC	G	C	G	C	cases	controls	cases	controls
**SS**																
Sookoian	40	94	NA	NA	NA	NA	NA	NA	42	38	63	125	52.5	33.6	48.5	66.4
Rotman	82	336	NA	NA	NA	NA	NA	NA	85	79	153	519	51.8	22.8	48.2	77.2
Hotta	64	575	19	26	19	104	296	175	64	64	504	646	50	43.8	50	56.2
Zain	33	198	NA	NA	NA	NA	NA	NA	23	43	95	301	35.0	24.0	65.0	76.0
Guichelaar	60	12	4	13	43	0	3	9	21	99	3	21	17.5	12.5	82.5	87.5
Verrijken	57	79	1	14	42	0	23	56	16	98	23	135	14.0	14.6	86.0	85.4
Kitamoto	51	1012	10	24	17	199	513	300	44	58	911	1113	43.1	45.0	56.9	55.0
Total	387	2306	34	77	121	303	835	540	295	479	1752	2860	38.1	38.0	61.9	62.0
**NASH**																
Sookoian	63	94	NA	NA	NA	NA	NA	NA	88	38	63	125	69.8	33.6	30.2	66.4
Rotman	438	336	NA	NA	NA	NA	NA	NA	431	445	153	519	49.2	22.8	45.8	77.2
Hotta	189	575	78	85	26	104	296	175	241	137	504	646	63.8	43.8	36.2	56.2
Zain	111	198	NA	NA	NA	NA	NA	NA	106	116	95	301	48.0	24.0	52.0	76.0
Guichelaar	72	12	8	28	36	0	3	9	44	100	3	21	30.6	12.5	69.4	87.5
Verrijken	151	79	16	69	66	0	23	56	101	201	23	135	33.4	14.6	66.6	85.4
Kitamoto	442	1012	187	183	72	199	513	300	557	327	911	1113	63.0	45.0	37.0	55.0
Total	1466	2306	289	365	200	303	835	540	1568	1364	1752	2860	53.5	38.0	46.5	62.0

SS: simple steatosis; NASH: nonalcoholic steatohepatitis.

**Table 5:** Association between PNPLA3 polymorphism and simple steatosis risk

Group	Study number(n)	NO. of cases/controls(n/n)	P_heterogeneity_	I^2^ (%)	Pooled OR (95%CI)	P value^a^
**SS**						
Allele contrast	7	774/4612	P < 0.0001	81	1.59 (1.02, 2.49)	P = 0.04
Dominant model	4	232/1678	P = 0.94	0	0.94 (0.66, 1.33)	P = 0.73
Recessive model	4	232/1678	P = 0.49	0	1.49 (0.97, 2.30)	P = 0.07
Additive model	4	155/843	P = 0.58	0	1.34 (0.82, 2.20)	P = 0.25
**Caucasian**						
Allele contrast	4	478/1042	P = 0.005	76	1.98 (1.05, 3.75)	P = 0.04
Dominant model	2	117/91	P = 0.71	0	0.93 (0.48, 1.82)	P = 0.84
Recessive model	2	117/91	P = 0.74	0	2.77 (0.30, 25.51)	P = 0.37
Additive model	2	90/65	P = 0.75	0	2.69 (0.29, 24.94)	P = 0.38
**Asian**						
Allele contrast	3	296/3570	P = 0.20	37	1.22 (0.89, 1.67)	P = 0.22
Dominant model	2	115/1587	P = 0.62	0	0.94 (0.62, 1.42)	P = 0.77
Recessive model	2	115/1587	P = 0.16	49	1.44 (0.93, 2.25)	P = 0.10
Additive model	2	65/778	P = 0.23	30	1.28 (0.77, 2.14)	P = 0.35

SS: simple steatosis.

a: Test for overall effect.

**Table 6:** Association between PNPLA3 Polymorphism and NASH risk

Group	Study number(n)	NO. of cases/controls(n/n)	P_heterogeneity_	I^2^ (%)	Pooled OR (95%CI)	P value^a^
**NASH**						
Allele contrast	7	2932/4612	P = 0.005	68	2.78 (2.24, 3.44)	P < 0.00001
Dominant model	4	854/1678	P = 0.63	0	2.44 (1.95, 3.04)	P < 0.00001
Recessive model	4	854/1678	P = 0.63	0	3.15 (2.58, 3.85)	P < 0.00001
Additive model	4	489/843	P = 0.49	0	4.44 (3.39, 5.82)	P < 0.00001
**Caucasian**						
Allele contrast	4	1448/1042	P = 0.59	0	3.40 (2.82, 4.09)	P < 0.00001
Dominant model	2	223/91	P = 0.95	0	3.11 (1.82, 5.33)	P < 0.0001
Recessive model	2	223/91	P = 0.38	0	10.33 (1.42, 75.06)	P = 0.02
Additive model	2	126/65	P = 0.36	0	14.28 (1.96, 103.92)	P = 0.009
**Asian**						
Allele contrast	3	1484/3570	P = 0.24	30	2.26 (1.93, 2.65)	P < 0.00001
Dominant model	2	631/1587	P = 0.38	0	2.33 (1.83, 2.96)	P < 0.00001
Recessive model	2	631/1587	P = 0.79	0	3.05 (2.49, 3.74)	P < 0.00001
Additive model	2	363/778	P = 0.41	0	4.22 (3.21, 5.55)	P < 0.00001

a: Test for overall effect.
