# Supplementary material for: Association Between Patatin-Like Phospholipase Domain Containing 3 Gene (PNPLA3) Polymorphisms and Nonalcoholic Fatty Liver Disease: A HuGE Review and Meta-Analysis
Source: Sci Rep. 2015 Mar 20;5:9284. doi: 10.1038/srep09284 (PMC4366950; doi:10.1038/srep09284)
Supplement: Supplementary Information [file srep09284-s1.pdf]

Association Between Patatin-Like Phospholipase Domain Containing 3 Gene (PNPLA3) Polymorphisms and Nonalcoholic Fatty Liver Disease: A HuGE Review and Meta-Analysis

Renfan Xu[1], Anyu Tao[1], Shasha Zhang[2], Youbin Deng[1], Guangzhi Chen[2].

[1]Department of Medical Ultrasound, Tongji Hospital, Tongji Medical College, Huazhong University of Science and Technology, Wuhan, People's Republic of China; [2]Department of Internal Medicine and Gene Therapy Center, Tongji Hospital, Tongji Medical College, Huazhong University of Science and Technology, Wuhan, People's Republic of China.

Table S1. Quality assessment of included studies.

First author	Representativeness of case-patients	Representativeness of controls	Ascertainment of NAFLD	Ascertainment of controls	Ascertainment of genotyping examination	Test for HWE	Association assessment	Total score
Kantartzis	1	1	2	2	0	2	2	10
Sookoian	1	1	2	2	0	2	2	10
Valenti 2010	1	2	2	2	0	2	2	11
Rotman	1	2	2	0	0	0	2	7
Speliotes	1	1	2	2	0	2	2	10
Goran	1	2	2	2	0	2	2	9
Lin 2011	1	2	2	2	0	2	2	11
Hotta	0	0	2	2	0	2	2	8
Wang	1	1	2	2	0	2	2	10
Petit	1	1	2	2	0	0	2	8
Zain	1	1	2	2	0	2	2	10
Kawaguchi	1	1	2	2	0	2	2	10
Valenti 2012	1	1	2	2	1	0	2	9
Li	1	1	2	2	0	2	2	10
Peng	1	1	2	2	1	2	2	11
Lin 2013	1	2	2	2	0	0	2	9
Guichelaar	1	1	2	2	0	0	2	8
Verrijken	1	1	2	2	0	2	2	10
Kitamoto	1	2	2	2	0	2	2	11
Musso et al.	1	2	2	2	0	2	2	11
Lin et al.	1	2	2	2	0	2	2	11
Niu et al.	0	2	2	2	0	2	2	10

Lee et al.	1	2	2	2	0	2	2	11

TableS2A: Sensitivity analysis of NAFLD (additive model).

Study omitted	I^2(%)	$P_{heterogeneity}$	Pooled OR(95%CI)	P value
Kantartzis, 2009	78	P<0.00001	3.51(2.63, 4.70)	P<0.00001
Valenti, 2010	77	P<0.00001	3.29(2.47, 4.38)	P<0.00001
Goran, 2010	77	P<0.00001	3.37(2.51,4.53)	P<0.00001
Lin, 2011	78	P<0.00001	3.43(2.55, 4.62)	P<0.00001
Hotta, 2010	78	P<0.00001	3.40(2.51,4.61)	P<0.00001
Wang, 2011	77	P<0.00001	3.54(2.63, 4.75)	P<0.00001
Kawaguchi, 2012	78	P<0.00001	3.50(2.56, 4.77)	P<0.00001
Valenti, 2012	78	P<0.00001	3.41(2.54, 4.58)	P<0.00001
Li, 2012	78	P<0.00001	3.40(2.52,4.58)	P<0.00001
Peng, 2012	76	P<0.00001	3.55(2.64, 4.77)	P<0.00001
Lin, 2013	77	P<0.00001	3.53(2.63, 4.75)	P<0.00001
Guichelaar, 2013	78	P<0.00001	3.41(2.57, 4.54)	P<0.00001
Verrijken, 2013	78	P<0.00001	3.36(2.53, 4.45)	P<0.00001
Kitamoto, 2013	78	P<0.00001	3.42(2.49, 4.68)	P<0.00001
Musso	78	P<0.00001	3.37(2.52, 4.52)	P<0.00001
Lin, 2014	77	P<0.00001	3.53(2.63, 4.75)	P<0.00001
Niu	40	0.05	2.90(2.42, 3.48)	P<0.00001
Lee	78	P<0.00001	3.49(2.60, 4.70)	P<0.00001

TableS2B: Sensitivity analysis of NASH (additive model).

Study omitted	I^2(%)	$P_{heterogeneity}$	Pooled OR(95%CI)	P value
Hotta, 2010	0	0.38	4.22(3.06, 5.81)	P<0.00001
Guichelaar, 2013	18	0.29	4.44(3.39, 5.82)	P<0.00001
Verrijken, 2013	0	0.48	5.74(3.52, 9.36)	P<0.00001
Kitamoto, 2013	0	0.71	4.23(3.22, 5.55)	P<0.00001

TableS2C: Sensitivity analysis of SS (additive model).

Study omitted	I^2(%)	$P_{heterogeneity}$	Pooled OR(95%CI)	P value
Hotta, 2010	0	0.61	1.04(0.50, 2.16)	P=0.92
Guichelaar, 2013	0	0.39	1.32(0.80, 2.19)	P=0.28
Verrijken, 2013	0	0.47	1.30(0.78, 2.15)	P=0.31
Kitamoto, 2013	0	0.87	1.77(0.93, 3.38)	P=0.08
